# Accelerated aging associated with cancer characteristics and treatments among breast cancer survivors

**DOI:** 10.18632/aging.206218

**Published:** 2025-03-07

**Authors:** Cong Wang, Jill B. De Vis, Kirsten Nguyen, Brigitte Jia, Mason Alford, Marjan Rafat, Bapsi Chakravarthy, Xiao-Ou Shu

**Affiliations:** 1 Division of Epidemiology, Department of Medicine, Vanderbilt University Medical Center, Nashville, TN 37235, USA; 2 Department of Radiation Oncology, Vanderbilt University Medical Center, Nashville, TN 37235, USA; 3 Vanderbilt University School of Medicine, Nashville, TN 37235, USA; 4 Department of Chemical and Biomolecular Engineering, Vanderbilt University, Nashville, TN 37235, USA; 5 Department of Biomedical Engineering, Vanderbilt University, Nashville, TN 37235, USA

**Keywords:** accelerated aging, PhenoAge, breast cancer, survivors

## Abstract

Breast cancer (BC) survivors may experience accelerated aging due to detrimental effects of BC and/or its treatments. Our study aims to evaluate Phenotypic Age Acceleration (PAA), a biological age measure, among BC patients and assess its associations with cancer characteristics and treatments. In this study including 1264 BC patients (age 54.7±11.7) and 429 cancer-free controls (age 49.9±12.4), we evaluated the differences in PAA (ΔPAA) by BC characteristics and treatments at multiple time points using linear mixed models. Overall, BC survivors had a higher PAA than controls at diagnosis (ΔPAA=3.73, *p*<0.001), 1-year (ΔPAA=1.68, *p*=0.001), and 10-year (ΔPAA=1.16, *p*=0.03) post-diagnosis. At 10-year post-diagnosis, stage III/IV (vs 0), intermediate- and high- (vs low-) grade BC were associated with a higher PAA of 4.48 (*p*<0.001), 1.26 (*p*=0.03), and 1.95 (*p*=0.001), respectively; triple-negative (vs hormone receptor+/HER2-) BC was associated with a lower PAA (ΔPAA=-1.96, *p*=0.004). Compared with patients receiving surgery with or without radiotherapy, higher PAA was observed at 1-year post-diagnosis among those receiving additional chemotherapy (ΔPAA=4.26, *p*<0.001) and at 10-year post-diagnosis for endocrine therapy (ΔPAA=2.89, *p*=0.001). In conclusion, BC patients had accelerated aging up to 10 years post-diagnosis, especially among those with stage III/IV and high/intermediate-grade BC, and receiving systemic treatment.

## INTRODUCTION

Breast cancer (BC) is the most commonly diagnosed cancer among women in the US and worldwide [1]. Early detection and effective treatments have made BC one of the most treatable cancers, with the five- and ten-year survival rates reaching 91% and 85%, respectively [1, 2] As a result, BC survivors are a large and fast-growing population, with over 4 million women with a history of BC living in the US [3]. Despite the success of current BC treatments in expanding the lifespan, there is concern that these treatments may have long-term detrimental effects on cancer survivors, with accumulating evidence of an increased rate of physical and cognitive decline among BC survivors than cancer-free women [4, 5]. These aging phenotypes indicate that BC survivors may experience accelerated aging.

Aging can be measured by quantitative molecular models based on biological age (BA) [6–11]. A few studies have investigated aging-related biomarkers among BC survivors, including DNA methylation (DNAm)-based BA metrics and p16INK4a, indicating a potential effect of chemotherapy, radiation therapy, and endocrine therapy on accelerated aging [12–14]. However, those studies were subject to small sample sizes (89 to 190 BC patients), lack of serial biomarker measures, and limited control for other treatments. Phenotypic Age Acceleration (PAA) is a recently established measure of aging based on chronological age (CA) and nine clinical blood chemistry markers, which are measured in the routine blood tests during patients’ clinic visits (except C-reactive protein [CRP]). Therefore, PAA is easily accessible in the clinical setting and can serve as an ideal cost-effective measure of aging with great translational significance [15]. PAA has been shown to be highly predictive of all-cause and cause-specific mortality among the general populations [10, 16].

The long-term aging trajectory among BC survivors and whether it is affected by cancer treatments are still unclear, partly due to lack of longitudinal measures of aging in this population. Our study aims to depict the biological aging trajectory among BC survivors and evaluate the associations of accelerated aging with BC tumor characteristics and treatments.

## RESULTS

### Participant characteristics

**Figure 1:**
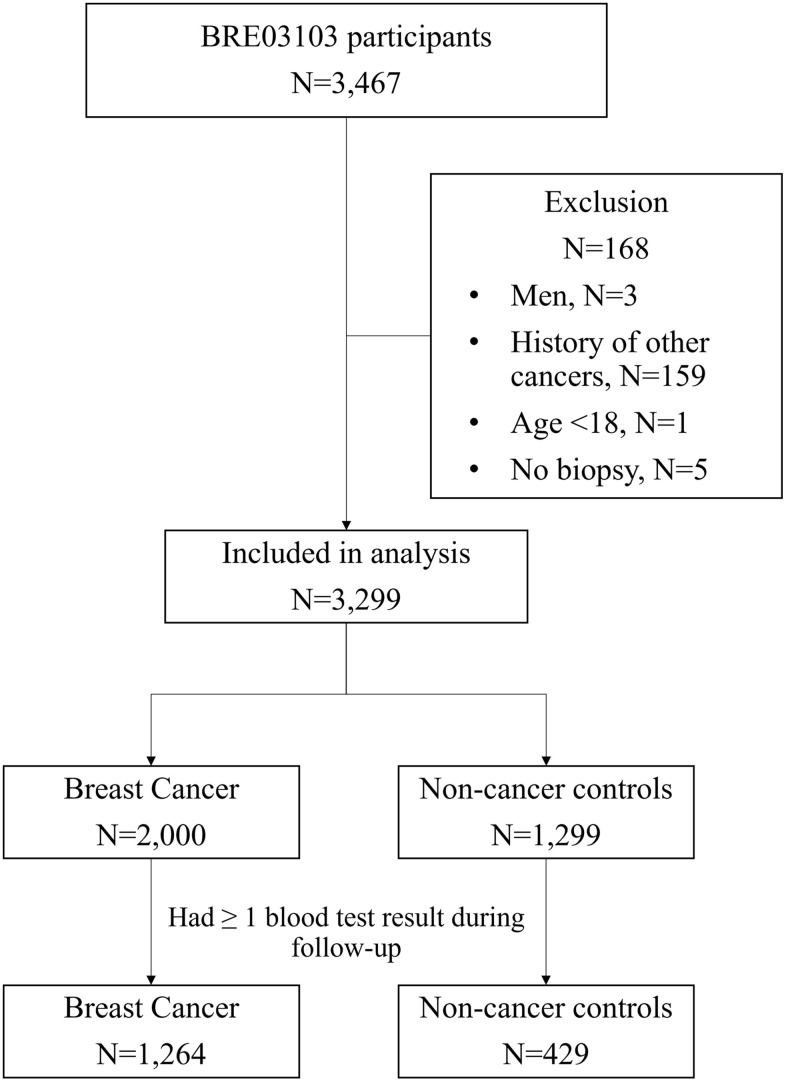
Study flowchart

A total of 1,264 BC patients and 429 controls were included in the study. A study flowchart is shown in Figure 1. BC cases were on average 4.8 years older than controls at biopsy, i.e., study enrollment (54.7 vs 49.9 years, *p*<0.001), had a 0.5-year longer follow-up (9.2 vs 8.7 years, *p*=0.01), and a higher proportion of death during the follow-up (8% vs 4%, *p*<0.001) (Table 1). Among BC patients, the majority were diagnosed at stage I (35%) or II (33%), and 17% were diagnosed at stage III/IV. There were 39% and 35% of the BC patients with intermediate- or high- grade BC, respectively. Almost half (45%) of the patients had hormone receptors (HR)+/ human epidermal growth factor 2 (HER2)- BC, 13% HER2+ BC, and 14% triple-negative BC (TNBC), i.e., HR-/HER2- BC. Most (89%) of the BC patients had surgery, 51% had radiation therapy, 60% had chemotherapy, 66% had endocrine therapy, 17% had targeted therapy, and 3% had immunotherapy. During a median follow-up of 9.1 years, 2% developed a second BC, and 20% had a recurrence or metastasis.

**Table 1:** Participant characteristics (*N*=1,693).

	**Breast cancer**	**Control**	***p***
	(N=1,264)	(N=429)	
Age at diagnosis/biopsy	54.7 ± 11.7	49.9 ± 12.4	<0.001
Follow-up duration (year)	9.2 ± 4.6	8.7 ± 3.2	0.01
Vital status at data request			
Alive	713 (56 %)	320 (75 %)	<0.001
Deceased	105 (8 %)	17 (4 %)	
Unknown	446 (35 %)	92 (21 %)	
Race			
White	1091 (86 %)	364 (85 %)	0.24
Black	113 (9 %)	49 (11 %)	
Other	60 (5 %)	16 (4 %)	
Charlson comorbidity index at diagnosis/biopsy	1.03 ± 1.51	0.84 ± 1.47	0.03
Charlson comorbidity index at latest follow-up	4.29 ± 3.17	3.00 ± 3.39	<0.001
**Tumor characteristics**			
Stage			
0	100 (8 %)		
I	447 (35 %)		
II	413 (33 %)		
III	152 (12%)		
IV	64 (5%)		
Unknown	88 (7 %)		
Grade			
Low	232 (18 %)		
Intermediate	499 (39 %)		
High	439 (35 %)		
Unknown	94 (7 %)		
Subtype			
HR+/HER2−	570 (45 %)		
HER2+	163 (13 %)		
Triple-negative	171 (14 %)		
Unknown	360 (28 %)		
**Treatments**			
Surgery	1124 (89 %)		
Radiation therapy	639 (51 %)		
Chemotherapy	759 (60 %)		
Alkylating	634 (50 %)		
Anthracyclines	469 (37 %)		
Antimetabolite	157 (12 %)		
Anti-microtubule/taxane	645 (51 %)		
Endocrine therapy	837 (66 %)		
Selective estrogen receptor modulators	437 (35 %)		
Aromatase inhibitors	634 (50 %)		
Targeted therapy	213 (17 %)		
Immunotherapy	40 (3 %)		
**Outcomes**			
Second breast cancer	20 (2 %)		
Recurrence/metastasis	250 (20 %)		

Mean±SD for *p*-values from t-test for continuous variables; n (%) and *p*-values from chi-square test for categorical variables.

### Associations of PAA with BC status and tumor characteristics

**Figure 2:**
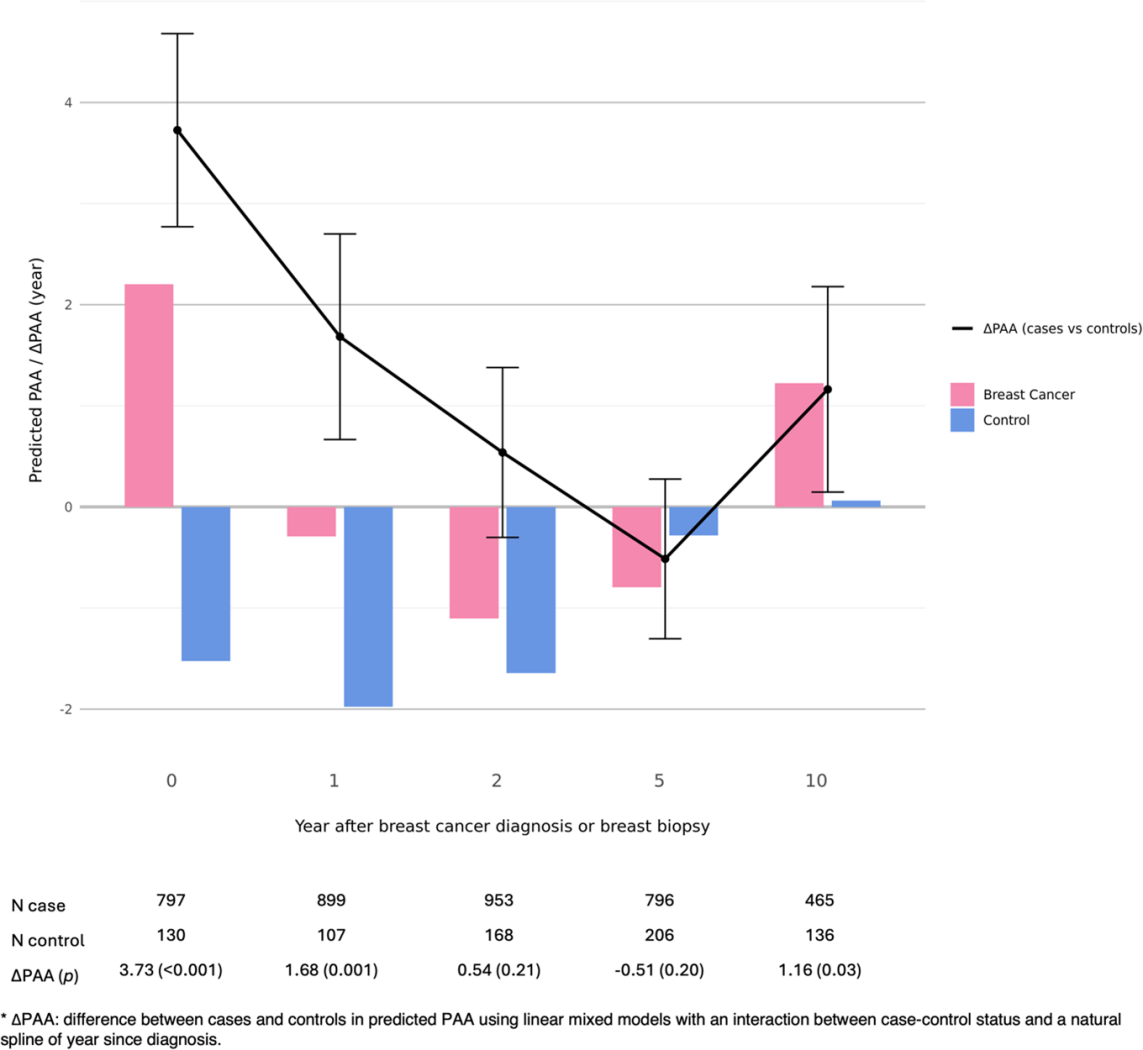
Difference in predicted PAA between breast cancer cases and controls by year since diagnosis.

The numbers of individuals with at least one PAA measure at each time point (i.e., at biopsy/diagnosis, 1-, 2-, 5-, and 10-years post-biopsy/diagnosis) were shown in Figure 2. The number of cases were larger (*N* range: 796 to 953) at time points during the first five years than at year 10 post-diagnosis (*N*=465). Compared with controls, BC patients had a significantly greater PAA of 3.73 (*p*<0.001) years at biopsy, 1.68 (*p*=0.001) years at year 1 post-biopsy and 1.16 (*p*=0.03) at year 10 post-biopsy (Figure 2). There were no significant differences in the predicted PAA between BC patients and controls at year 2 and 5 post-biopsy.

The patterns of PAA among BC patients differed by age at diagnosis. Among BC cases, compared to those diagnosed before 45, patients who were diagnosed at 65 or older had a lower PAA at diagnosis (ΔPAA=-2.33, *p*<0.001), but a higher PAA at year 2, 5, and 10 post-diagnosis, with ΔPAA of 1.53 (*p*=0.004), 1.62 (*p*=0.003), and 1.31 (*p*=0.047), respectively (Table 2).

**Table 2:** Association of PAA with patient and tumor characteristics among breast cancer patients (*N*=1,264).

**Tumor characteristics**	***N***	**At diagnosis**		**Year 1**		**Year 2**		**Year 5**		**Year 10**	
		**ΔPAA**	***p***	**ΔPAA**	***p***	**ΔPAA**	***p***	**ΔPAA**	***p***	**ΔPAA**	***p***
Age at diagnosis											
<45	247	Reference		Reference		Reference		Reference		Reference	
45-54	383	0.51	0.376	0.83	0.106	0.87	0.074	0.41	0.398	**-1.41**	**0.01**
55-64	379	-0.67	0.257	-0.96	0.062	-0.12	0.8	**1.02**	**0.04**	**1.47**	**0.008**
≥65	255	**-2.33**	**<0.001**	0.72	0.203	**1.53**	**0.004**	**1.62**	**0.003**	**1.31**	**0.047**
Stage											
0	100	Reference		Reference		Reference		Reference		Reference	
I	447	1.01	0.268	0.97	0.249	-0.88	0.237	**-1.56**	**0.03**	**-1.56**	**0.044**
II	413	**2.82**	**0.002**	**2.20**	**0.009**	-0.40	0.592	**-1.84**	**0.009**	**-1.59**	**0.042**
III/IV	216	**4.80**	**<0.001**	**2.96**	**0.001**	**2.78**	**<0.001**	**4.34**	**<0.001**	**4.48**	**<0.001**
Grade											
Low	232	Reference		Reference		Reference		Reference		Reference	
Intermediate	499	0.35	0.584	0.55	0.295	0.84	0.082	0.49	0.308	**1.26**	**0.03**
High	439	**2.08**	**0.001**	**3.02**	**<0.001**	**2.39**	**<0.001**	**1.28**	**0.008**	**1.95**	**0.001**
Subtype											
HR+/HER2-	570	Reference		Reference		Reference		Reference		Reference	
HER2+	163	-0.12	0.834	**2.27**	**<0.001**	**2.56**	**<0.001**	**1.52**	**0.003**	0.42	0.494
Triple-negative	171	0.12	0.843	**3.46**	**<0.001**	**1.93**	**<0.001**	0.17	0.753	**-1.96**	**0.004**

Difference in Phenotypic Age Acceleration (PAA) estimated using linear mixed models with an interaction between exposures and a natural spline of year since diagnosis.

Tumor characteristics were associated with PAA during the 10-year follow-up. Stage III/IV BC was associated with an increased PAA compared to stage 0 BC throughout the 10 years of follow-up, with ΔPAA of 4.80 (*p*<0.001) at diagnosis and increasing from 2.96 (*p*=0.001) at year 1 to and 4.48 (*p*<0.001) at year 10 post-diagnosis. A similar pattern was observed among high-grade BC patients, where a positive ΔPAA observed throughout the 10 years after diagnosis, with a peak of 3.02 (*p*<0.001) at year 1 post-diagnosis which was attenuated to 1.95 (*p*=0.001) at year 10 post-diagnosis, compared to patients with low-grade BC. Compared with HR+/HER2- BC patients, HER2+ patients had an increased PAA during 1 to 5 years post-diagnosis (ΔPAA=2.27, 2.56, 1.52, respectively, all *p*<0.01), and TNBC patients had an increased PAA during 1 to 2 years post-diagnosis (ΔPAA=3.46, 1.93, respectively, both *p*<0.001) which was reverted to a negative PAA at year 10 post-diagnosis (ΔPAA=-1.96, *p*=0.004).

### Associations of PAA with BC treatments

Among 1,236 BC patients who received at least one type of treatment, treatment type was associated with PAA during the 10 years of follow-up, after mutual adjustment for other treatments (Supplementary Table 1). At year 10 post-diagnosis, a positive association with PAA was observed for endocrine therapy (ΔPAA=1.62, *p*=0.001), while surgery (ΔPAA=-6.91, *p*<0.001) and radiation therapy (ΔPAA=-1.86, *p*<0.001) were associated with a lower PAA; no significant association was observed for chemotherapy (ΔPAA=-0.22, *p*=0.65) and targeted therapy (ΔPAA=0.07, *p*=0.92).

When the treatment was analyzed in combination, receiving radiation in addition to surgery was not associated with PAA increase during follow-up, compared with surgery only (Supplementary Table 2). Therefore, we re-grouped the treatment combinations to evaluate the association between treatment patterns and PAA (Table 3). After adjustment for cancer stage, grade, and subtype, patients receiving surgery (+/- radiation therapy), those with treatment combinations involving chemotherapy had an increased PAA at year 1 post-diagnosis, with ΔPAA of 4.26 (*p*<0.001) for receiving additional chemotherapy, and ΔPAA of 2.88 (*p*=0.01) for receiving additional chemotherapy, endocrine therapy, and targeted therapy (CT+ET+TT).

**Table 3:** Association between PAA and breast cancer treatments (*N*=1,236).

**Treatment**	***N***	**Year 1**		**Year 2**		**Year 5**		**Year 10**	
		**ΔPAA**	***p***	**ΔPAA**	***p***	**ΔPAA**	***p***	**ΔPAA**	***p***
**Treatment combination^1^**									
Surgery(+radiation)	116	Reference		Reference		Reference		Reference	
Surgery(+radiation)+chemo	171	**4.26**	**<0.001**	1.13	0.277	-0.41	0.686	0.92	0.399
Surgery(+radiation)+endocrine	326	0.32	0.739	-1.03	0.226	-0.45	0.58	**2.89**	**0.001**
Surgery(+radiation)+chemo+endocrine	327	1.38	0.164	-1.19	0.179	-1.38	0.102	1.32	0.152
Surgery(+radiation)+chemo+endocrine+targeted	106	**2.88**	**0.012**	0.65	0.539	0.13	0.901	**2.52**	**0.028**
Other	190	1.67	0.129	0.64	0.523	**1.91**	**0.05**	**4.88**	**<0.001**
**Medication^2^**	*N* (yes/no)								
Chemotherapy									
Alkylating	634/602	**1.77**	**0.002**	0.02	0.968	**-1.65**	**0.003**	**-2.44**	**<0.001**
Anthracyclines	469/767	**1.52**	**0.002**	-0.04	0.938	**-2.08**	**<0.001**	**-2.65**	**<0.001**
Antimetabolite	157/1,079	**-1.31**	**0.01**	**2.22**	**<0.001**	**5.41**	**<0.001**	**6.91**	**<0.001**
Anti-microtubule/taxane	645/591	1.02	0.053	0.13	0.793	-0.16	0.756	-0.60	0.278
Endocrine therapy									
Selective estrogen receptor modulators	437/799	**-1.25**	**0.001**	**-1.23**	**<0.001**	**-1.15**	**0.001**	-0.79	0.057
Aromatase inhibitors	634/602	**-0.86**	**0.029**	-0.71	0.062	0.27	0.48	**1.69**	**<0.001**

Analysis among breast cancer patients receiving at least one type of treatment; ^1^Difference in PAA estimated using linear mixed models with an interaction between exposures and a natural spline of year since diagnosis, with adjustment for cancer stage, grade, and subtype; ^2^Difference in PAA estimated using linear mixed models with an interaction between exposures and a natural spline of year since diagnosis, with adjustment for cancer stage, grade, subtype, other treatments.

At year 10 post-diagnosis, treatment combinations involving endocrine therapy was associated with an increased PAA, with ΔPAA of 2.89 (*p*=0.001) for receiving additional endocrine therapy, and ΔPAA of 2.52 (*p*=0.03) for receiving additional CT+ET+TT. The mixture of other treatment combinations was associated with an increased PAA at all time points.

We further examined the associations between PAA and specific anti-cancer drugs used for BC (Table 3). The associations of PAA with most chemotherapy agents (alkylating agents, anthracyclines, and anti-microtubule agents/taxane) diminished over time, reverting to negative or null at year 10 post-diagnosis, with the respective ΔPAA of alkylating agents, anthracyclines, and anti-microtubule agents/taxane of -2.44 (*p*<0.001), -2.65 (*p*<0.001), and -0.60, (*p*=0.28) at year 10. Antimetabolites, on the other hand, showed the opposite association, where a positive association was observed after 2 years post-diagnosis, which further increased to 6.91 years higher PAA (*p*<0.001) at year 10 compared to those not receiving antimetabolites. The association between endocrine therapy and PAA at year 10 post-diagnosis is potentially driven by aromatase inhibitors (AIs) (ΔPAA=1.69, *p*<0.001), while selective estrogen receptor modulators (SERMs) were associated with a non-significant decrease in PAA (ΔPAA=-0.79, *p*=0.06).

In the sensitivity analysis including 776 BC patients and 429 cancer-free control women with PAA measures both at baseline and during follow-up, some associations attenuated to null. BC patients had an increased PAA only at diagnosis (ΔPAA=3.90, *p*<0.001) and year 1 post-diagnosis (ΔPAA=1.85, *p*<0.001), but not at year 10 post diagnosis (ΔPAA=0.46, *p*=0.39). Stage III/IV BC was associated with an increase in PAA only at year 5 post-diagnosis (ΔPAA=3.04, *p*=0.03), but not at other time points (Table 4). The association between PAA and tumor grade remained largely unchanged. HER2+ BC and TNBC were associated with an increase in PAA during year 1 to 2 post-diagnosis. After adjustment for cancer stage, grade, and subtype, no significant associations between PAA during follow-up and treatment patterns were observed, potentially due to the limited sample size (Table 5).

**Table 4:** Association between PAA and tumor characteristics among breast cancer patients in sensitivity analysis (*N*=776).

**Tumor characteristics**	***N***	**At diagnosis**		**Year 1**		**Year 2**		**Year 5**		**Year 10**	
		**ΔPAA**	***p***	**ΔPAA**	***p***	**ΔPAA**	***p***	**ΔPAA**	***p***	**ΔPAA**	***p***
Age at diagnosis											
<45	162	Reference		Reference		Reference		Reference		Reference	
45-54	255	0.58	0.349	0.53	0.358	0.55	0.324	0.19	0.738	**-1.43**	**0.035**
55-64	226	-0.03	0.963	-0.67	0.254	-0.16	0.786	**2.12**	**<0.001**	1.26	0.075
≥65	133	**-2.18**	**0.003**	1.10	0.101	**1.52**	**0.020**	**1.80**	**0.008**	1.46	0.096
Stage											
0	19	Reference		Reference		Reference		Reference		Reference	
I	231	-0.67	0.627	-0.52	0.708	-1.92	0.147	**-2.89**	**0.033**	**-3.76**	**0.013**
II	330	0.71	0.597	0.85	0.533	-1.21	0.355	**-3.07**	**0.022**	**-4.42**	**0.003**
III/IV	183	2.57	0.061	1.34	0.332	1.83	0.169	**3.04**	**0.026**	0.60	0.696
Grade											
Low	112	Reference		Reference		Reference		Reference		Reference	
Intermediate	300	0.40	0.584	0.88	0.178	**1.43**	**0.020**	0.56	0.363	**1.67**	**0.032**
High	338	**1.72**	**0.016**	**3.14**	**<0.001**	**2.79**	**<0.001**	0.87	0.157	**1.80**	**0.019**
Subtype											
HR+/HER2-	350	Reference		Reference		Reference		Reference		Reference	
HER2+	141	-0.73	0.235	**2.21**	**<0.001**	**2.51**	**<0.001**	1.05	0.061	0.08	0.918
Triple-negative	142	-0.56	0.363	**3.33**	**<0.001**	**1.84**	**0.001**	-0.78	0.175	-1.09	0.162

Difference in PAA estimated using linear mixed models with an interaction between exposures and a natural spline of year since diagnosis.

**Table 5:** Association between PAA and breast cancer treatments in the sensitivity analysis (*N*=774).

**Treatment**	***N***	**Year 1**		**Year 2**		**Year 5**		**Year 10**	
		**ΔPAA**	***p***	**ΔPAA**	***p***	**ΔPAA**	***p***	**ΔPAA**	***p***
**Treatment combination^1^**									
Surgery(+radiation)	16	Reference		Reference		Reference		Reference	
Surgery(+radiation)+chemo	139	3.58	0.053	0.04	0.983	-1.12	0.539	-2.46	0.238
Surgery(+radiation)+endocrine	135	-0.67	0.702	-2.02	0.242	-0.74	0.667	-0.32	0.874
Surgery(+radiation)+chemo+endocrine	271	-0.04	0.982	-2.77	0.105	-2.84	0.095	-2.74	0.160
Surgery(+radiation)+chemo+endocrine+targeted	96	1.42	0.437	-0.97	0.593	-1.70	0.348	-0.73	0.725
Other	117	0.93	0.607	-0.10	0.954	1.09	0.543	0.12	0.952
**Medication^2^**	*N* (yes/no)								
Chemotherapy									
Alkylating	534/240	**1.70**	**0.008**	-0.33	0.605	**-2.31**	**<0.001**	**-2.89**	**<0.001**
Anthracyclines	393/381	**1.38**	**0.007**	-0.09	0.860	**-2.77**	**<0.001**	**-2.50**	**<0.001**
Antimetabolite	97/677	-0.64	0.286	**2.88**	**<0.001**	**7.28**	**<0.001**	**6.34**	**<0.001**
Anti-microtubule/taxane	552/222	0.51	0.420	-0.25	0.682	**-1.35**	**0.030**	**-1.94**	**0.005**
Endocrine therapy									
Selective estrogen receptor modulators	285/489	**-1.89**	**<0.001**	**-1.48**	**0.001**	**-1.60**	**<0.001**	0.04	0.942
Aromatase inhibitors	424/350	-0.88	0.065	-0.71	0.129	0.63	0.185	0.92	0.103

^1^Difference in PAA estimated using linear mixed models with an interaction between exposures and a natural spline of year since diagnosis, with adjustment for cancer stage, grade, and subtype; ^2^Difference in PAA estimated using linear mixed models with an interaction between exposures and a natural spline of year since diagnosis, with adjustment for cancer stage, grade, subtype, other treatments, and other drugs of the treatment type.

We found that higher baseline PAA was associated with higher risk for all-cause mortality among 797 BC patients with available baseline measures (hazard ratio [HR]=1.05, 95% CI=1.01, 1.08). This association remained after removing 249 patients without confirmed vital status (HR=1.05, 95% CI=1.01, 1.08) and further adjustment for BC stage, grade, and subtype (HR=1.04, 95% CI=1.00, 1.07).

## DISCUSSION

In this clinic-based cohort of BC patients and cancer-free women, we observed age acceleration up to 10 years post-diagnosis. Tumor characteristics, including advanced stage, high tumor grade, and HER2+ as well as triple-negative subtypes, were associated with higher age acceleration. Systemic treatment was associated with accelerated aging, especially chemotherapy in the short term and endocrine therapy in the long term. Antimetabolites and AIs were associated with long-term accelerated aging. Higher age acceleration at BC diagnosis was associated with an increased risk for all-cause mortality among BC survivors.

Emerging evidence indicates that BC survivors might experience accelerated aging, as measured by aging-related diseases, physical and cognitive function decline, DNAm-based biomarkers and p16INK4a [4, 5, 12–14, 17]. Previous research demonstrated that exposure to chemotherapy and/or radiation was associated with markers of cellular aging, including higher DNA damage and lower telomerase activity, 3-6 years post-diagnosis among 94 BC survivors [18]. However, due to the small sample size and no serial sample collection, the trajectories of these biomarker changes and treatment-specific effects were unclear.

To our knowledge, our study was the first to investigate the blood chemistry marker based PAA among BC survivors. Several studies have investigated the epigenetic BA metrics, including DNAm-based PAA, among BC survivors [13, 14]. In a study of 417 women including 190 BC survivors by Kresovich et al., BC survivors had a higher DNAm-based PAA compared to cancer-free women (β=0.13, 95% CI=0.00, 0.26, *p*=0.04) at approximately 4 years post-diagnosis, and radiation therapy was significantly associated with an increase in DNAm-based PAA (β=0.39, 95% CI=0.19, 0.59, *p*<0.001) [13]. In our study, however, radiation therapy had a null or inverse association with PAA whereas treatments with chemotherapy as well as endocrine therapy were associated with higher PAA. Similar to our results, Kresovich’s study showed a time-dependent positive association of BA acceleration seen with endocrine therapy, which was only significant with the BA metric DunedinPACE measured beyond 4 years post-diagnosis, indicating the relatively long-term effect of endocrine therapy [13]. In another study of 89 BC survivors by Rentscher et al. Chemotherapy and endocrine therapy were associated with multiple increased BA metrics at approximately 2 to 4 years post-diagnosis [14]. These studies were limited by small sample sizes, lack of serial measures of BA, and/or lack of adjustment for other treatments, thus hindering the investigation of the longer-term aging trajectories and treatment-specific effects on accelerated aging.

The mechanisms underlying the age acceleration among BC patients involve intricate cellular and molecular processes associated with BC and related treatment. The age acceleration could be partly attributed to the damage and dysfunction in normal cells induced by chemotherapy treatments, including telomere attrition, mitochondrial dysfunction, genomic instability, epigenetic alterations, cellular senescence, and chronic inflammation [4, 19–21]. Particularly, antimetabolites-involved regimen of cyclophosphamide, methotrexate, and fluorouracil (CMF) have been associated with elevated levels of inflammatory markers and lower cognitive performance at 20 years after chemotherapy [19]. Our findings of association between antimetabolites and accelerated aging becoming stronger over time also emphasized its potential long-term adverse effects. In addition, cumulative evidence has suggested that endocrine therapy may contribute to age acceleration by perturbing hormonal homeostasis and interfering with the beneficial effects of estrogen on aging, resulting in biological aging features, such as stem cell exhaustion, genomic instability, altered intercellular communication, and mitochondrial dysfunction [22]. Menopause might be important in understanding the effects of endocrine therapy on age acceleration among BC patients. However, we were unable to evaluate it because the information on age at menopause was not precisely recorded in the electronic health record (EHR).

This is the first large study with 10 years of follow-up to evaluate PAA among BC survivors. With repeated measures of PAA and detailed information of cancer diagnosis and treatments, we evaluated the aging trajectory from BC diagnosis to 10 years post-diagnosis and identified risk factors for accelerated aging among BC survivors.

We would like to point out that rather than measuring the exact aging trajectories among the same group of BC survivors over time, our study assessed the age acceleration among a group of BC survivors across different time points post-cancer diagnosis. Because patients with a higher PAA were more likely at a higher risk of recurrence and/or mortality, the loss to follow-up rate would be higher among these patients. Such survival bias needs to be considered in the interpretation of our findings. For example, the attenuation of biological age acceleration over time could be two-fold: 1) the effects of BC characteristics and treatments might be greater at the time of diagnosis or active treatment, compared with other time points; and 2) BC survivors with a higher PAA were more likely to be lost to follow-up due to recurrence and/or mortality, and those who were included in the later follow-up were more robust (had a lower PAA) than the BC patients overall. Similar caution should apply to the finding of the inverse associations of alkylating agents and anthracyclines with 10-year PAA. The different aging trajectories among younger (age <45) and older (age ≥65) BC patients could potentially reflect the more aggressive cancer treatment the younger patients received, their ability to recover, and the survival bias.

The limitations of the study include the selection bias of the participants, particularly in the recruitment of cancer-free controls from the same clinic as BC patients. Many controls underwent needle biopsy due to suspicious breast lesions, different from the cancer-free women from the general population, potentially leading to an underestimation of case-control differences. Of note, BC patients in our study population were younger and more likely to be diagnosed with triple-negative BC, compared with the US national data [3]. In addition, there is heterogeneity in the number of measures of PAA among the participants. A greater number of PAA measures could be indicative of more frequent hospital visits due to complex health conditions. While efforts were made to exclude PAA measured during inpatient or Emergency Department visits, not all acute conditions that could induce temporal alterations in blood chemistry markers could be excluded. Despite utilizing a mixed model to account for the data structure, our results primarily reflect the aging trajectory of patients with a sufficient number of blood measures over an extended duration of follow-up. Limited by the clinical setting of the study, our calculated PAA differed from the original PAA developed by Levine et al. [10] in that we re-weighted the biomarkers after dropping the highly missing variable, CRP, which is tested only when the patients were suspected to have an infection. However, the updated PAA had a 0.995 correlation with the original PAA, suggesting a minimal effect of dropping CRP from the PAA estimation. Additionally, we were underpowered in evaluating the effects of immunotherapy on accelerated aging given the small number of BC patients receiving such treatment. Lack of information on demographics, lifestyles, age at menopause, and self-reported aging-related outcomes from the study participants limited our ability to evaluate the influence of other factors on accelerated aging among BC survivors. Lastly, because the vital status and date of death are not always available in the EHR, the PAA-mortality association is likely to be underestimated in our study.

In summary, this study provides evidence of accelerated aging among BC survivors and identified the high-risk populations with certain tumor characteristics and/or treatments. However, it remains challenging to fully disentangle the effects of each characteristic or treatment on accelerated aging, as some associations with cancer characteristics may be mediated by the treatment. Our findings call for future research among cancer survivors to integrate demographics, lifestyles, and self-reported aging-related outcomes and enroll BC survivors with modern treatment, which could further our understanding of the long-term challenges during survivorship. Along with the growing evidence of accelerated aging among cancer survivors, such research could ultimately be translated into guidance for care, aiming to improve quality of life and overall health of the large and growing population of cancer survivors.

## MATERIALS AND METHODS

### Study participants

From 2004 to 2021, participants were enrolled into a breast tissue/body fluids repository project at Vanderbilt-Ingram Cancer Center. Eligibility criteria included ≥18 years of age and having a breast lesion suspicious for cancer (controls) or a known diagnosis of BC (cases). All participants provided consent for future research, including accessing their EHR data and further contact. In the current study, we included participants with no prior history of cancer (other than BC), and with at least one measure of PAA during follow-up (0.5 to 15 years after biopsy). We excluded participants who died within two years after biopsy and censored participants’ information at the time of diagnosis of a second BC, recurrence, metastasis, or two months before death. Participant demographics and medical information were obtained from the cancer registry and EHR.

### Assessment of exposures

Exposures in this study included BC status (case vs control), tumor characteristics, and cancer-related treatments. Tumor characteristics included BC stage (0-IV), grade (low, intermediate, and high), and BC subtype based on HR and HER2 status (HR+/HER2-, HER2+, and TNBC). BC treatment modalities included surgery, radiation therapy, chemotherapy, endocrine therapy, targeted therapy, and immunotherapy. Chemotherapy agents were categorized into alkylating agents, anthracyclines, antimetabolites, and anti-microtubules/taxanes. Endocrine therapy was categorized into SERMs and AIs.

### Assessment of outcomes

Phenotypic Age (PhenoAge) is a biological aging clock, calculated using CA and nine blood biomarkers (prevalence in all lab records, %) [albumin (80%), creatinine (93%), glucose (92%), CRP (16%), lymphocyte percent (65%), mean cell volume (83%), red blood cell distribution width (83%), alkaline phosphatase (81%), and white blood cell count (83%)]. It was developed and validated in nationally representative US samples to predict mortality [10]. In this study, PhenoAge was calculated based on the blood test results from the patients’ clinic visits (excluding inpatient and Emergency Department visits) from the time of initial biopsy to 15 years after biopsy. Due to the low availability of CRP in our data, we modified the PhenoAge following the same strategy as its original development [10] using the 8 remaining available biomarkers. The participants need to have all the 8 biomarkers measured on the same date to have a PhenoAge estimate. The modified PhenoAge had a 0.995 Pearson correlation coefficient with the original PhenoAge and resulted in the same hazard ratio for all-cause mortality as the original PhenoAge in NHANES IV women (HR=1.07, 95% CI=1.06, 1.08).

The modified PhenoAge formula is as follows:


$$Phenotypic\;Age=143.59+\frac{\ln\;[-0.00645\times \ln\;(1-M)]}{0.08555}$$

Where:


$$M=1-\exp\;(\frac{-1.52078\times \exp\;(xb)}{0.0077047})$$

and:


$$\begin{array}{l} xb=-17.676-0.0344\times Albumin+0.0079\\ \;\;\;\;\;\;\;\times Creatinine+0.1120\times Glucose\\ -0.0111\times Lymphocyte\;Percent+0.0283\\ \;\;\;\;\;\;\;\times Mean\;Cell\;Volume\\ +0.2072\times Red\;Cell\;Distribution\;Width\\ +0.00202\times Alkaline\;Phosphatase+0.0737\\ \;\;\;\;\;\;\;\;\;\times White\;Blood\;Cell\;Count\\ +0.0776\times Chronological\;Age \end{array}$$

PAA was then calculated as the difference between PhenoAge and CA. Each participant could have multiple measures of PAA during follow-up. We also calculated PAA as residuals of the linear model that regresses PhenoAge and CA (results in Supplementary Tables 3, 4).

### Statistical analysis

The associations between PAA and BC status, age at diagnosis, tumor characteristics (stage, grade, and subtype), and treatments were evaluated using linear mixed models, with the time interval between diagnosis and PAA assessments and its interaction with the exposure as covariates, and subject as a random effect. The time interval between diagnosis and PAA assessments was treated as a natural spline in the models with 3 knots, i.e., at year 1, 2, and 5 post-diagnosis. The effects of treatment modalities were evaluated among BC cases only with additional adjustment for tumor characteristics and mutual adjustment for other treatments. The associations between PAA and systemic chemotherapy medications were evaluated among BC cases with adjustment for age, tumor characteristics, treatment modalities, and other drugs used in the same treatment modality. The difference between predicted PAA derived from the linear mixed models and chronological age was presented in ΔPAA (in years) at diagnosis and at year 1, 2, 5, and 10 post-diagnosis. Sensitivity analysis was conducted by restricting BC patients to those who had PAA measures both at baseline and during follow-up, i.e., excluding patients without PAA measures around diagnosis (between 1 year prior to diagnosis to 0.5 years after diagnosis). Further, we evaluated the association between baseline PAA and all-cause mortality among BC patients who had available PAA measures using Cox proportional hazards models. Statistical significance was determined using a two-sided *p*-value <0.05. All analysis was performed in R version 4.2.1.

## Supplementary Material

Supplementary Tables
