# Supplementary material for: Accelerated aging associated with cancer characteristics and treatments among breast cancer survivors
Source: Aging (Albany NY). 2025 Mar 7;17(3):643–56. doi: 10.18632/aging.206218 (PMC11984420; doi:10.18632/aging.206218)
Supplement: Supplementary Tables [file aging-17-206218-s001.pdf]

SUPPLEMENTARY TABLES

Supplementary Table 1. Association between PAA and breast cancer treatments (*N*=1,236).

Treatment	*N* yes/ *N* no	At diagnosis Δ PAA	At diagnosis *p*	Year 1 Δ PAA	Year 1 *p*	Year 2 Δ PAA	Year 2 *p*	Year 5 Δ PAA	Year 5 *p*	Year 10 Δ PAA	Year 10 *p*
Surgery	1,124 /112	**1.9**	**0.03**	0.47	0.549	**-2.59**	**0.001**	**-5.64**	**<0.001**	**-6.91**	**<0.001**
Radiation therapy	639/597	0.79	0.056	0.62	0.083	-0.47	0.174	**-1.78**	**<0.001**	**-1.86**	**<0.001**
Chemotherapy	759/477	**4.83**	**<0.001**	**1.81**	**<0.001**	**0.84**	**0.05**	0.11	0.79	-0.22	0.647
Hormone therapy	837/399	**1.52**	**0.003**	**-1.13**	**0.015**	-0.79	0.082	0.11	0.813	**1.62**	**0.001**
Targeted therapy	213/1,023	-1.08	0.105	-0.29	0.647	0.01	0.993	-0.08	0.894	0.07	0.923

Difference in PAA estimated using linear mixed models with an interaction between exposures and a natural spline of year since diagnosis, with adjustment for cancer stage, grade, subtype, and mutual adjustment for other treatments.

Supplementary Table 2. Association between PAA and breast cancer treatment pattern (*N*=1,236).

Treatment	*N*	At diagnosis Δ PAA	At diagnosis *p*	Year 1 Δ PAA	Year 1 *p*	Year 2 Δ PAA	Year 2 *p*	Year 5 Δ PAA	Year 5 *p*	Year 10 Δ PAA	Year 10 *p*
Surgery only	67	Reference		Reference		Reference		Reference		Reference	
Surgery+radiation	49	-0.41	0.843	0.61	0.726	-0.87	0.561	-2.4	0.077	1.1	0.459
Surgery+chemo	69	**5.91**	**<0.001**	**4.83**	**0.001**	1.68	0.209	-0.21	0.865	2.44	0.078
Surgery+hormone	140	1.34	0.367	0.44	0.738	-1.6	0.162	-1.83	0.089	**3.81**	**0.002**
Surgery+radiation+ hormone	186	0.86	0.564	0.51	0.691	-1.32	0.234	-1.14	0.27	**3.07**	**0.007**
Surgery+chemo+ hormone	142	**6.58**	**<0.001**	1.43	0.277	-1.5	0.195	-1.5	0.171	**2.66**	**0.026**
Surgery+radiation+ chemo	102	**3.99**	**0.01**	**3.85**	**0.009**	-0.34	0.798	-2.4	0.06	0	0.999
Surgery+radiation+ chemo+hormone	185	**6.84**	**<0.001**	1.71	0.186	-1.67	0.141	**-2.99**	**0.005**	1.09	0.346
Surgery+radiation+ chemo+hormone+ targeted	66	**6.22**	**<0.001**	**2.98**	**0.04**	-0.19	0.883	-1.46	0.249	**2.77**	**0.046**
Other	230	**3.81**	**0.009**	2.14	0.109	0.29	0.806	0.75	0.504	**4.97**	**<0.001**

Difference in PAA estimated using linear mixed models with an interaction between exposures and a natural spline of year since diagnosis, with adjustment for cancer stage, grade, and subtype.

Supplementary Table 3. Association between PAA as residuals and clinical characteristics among breast cancer patients (*N*=1,264).

Clinical characteristics	*N*	At diagnosis		Year 1		Year 2		Year 5		Year 10	
		Δ PAA	*p*	Δ PAA	*p*	Δ PAA	*p*	Δ PAA	*p*	Δ PAA	*p*
Cases vs controls	1,264	**2.09**	**<0.001**	0.28	0.423	-0.42	0.092	**-0.88**	**<0.001**	0.25	0.373
Age at diagnosis											
<45	247	Reference		Reference		Reference		Reference		Reference	
45-54	383	-0.12	0.714	0.2	0.381	0.31	0.095	-0.02	0.898	**-0.6**	**0.01**
55-64	379	-0.69	0.052	**-0.66**	**0.005**	-0.14	0.457	**0.44**	**0.012**	**0.55**	**0.015**
≥65	255	**-2.53**	**<0.001**	0.28	0.29	**0.66**	**0.002**	0.39	0.089	0.44	0.193
Stage											
0	100	Reference		Reference		Reference		Reference		Reference	
I	447	**1.27**	**0.028**	0.92	0.054	-0.41	0.268	**-0.68**	**0.023**	-0.21	0.563
II	413	**1.94**	**<0.001**	**0.92**	**0.049**	**-1.01**	**0.006**	**-1.76**	**<0.001**	-0.5	0.186
III/IV	216	**1.41**	**0.013**	-0.41	0.383	-0.39	0.282	**0.64**	**0.032**	**0.84**	**0.037**
Grade											
Low	232	Reference		Reference		Reference		Reference		Reference	
Intermediate	499	-0.24	0.561	-0.03	0.906	0.12	0.572	-0.15	0.443	0.11	0.688
High	439	-0.24	0.548	0.4	0.136	0.03	0.876	**-0.67**	**0.001**	0.07	0.787
Subtype											
HR+/HER2-	570	Reference		Reference		Reference		Reference		Reference	
HER2+	163	**-1.39**	**<0.001**	**0.6**	**0.003**	**0.84**	**<0.001**	-0.05	0.802	-0.23	0.441
Triple-negative	171	**-1.34**	**<0.001**	**0.92**	**<0.001**	0.05	0.825	**-1.06**	**<0.001**	**-1.65**	**<0.001**

Difference in PAA estimated using linear mixed models with an interaction between exposures and a natural spline of year since diagnosis.

Supplementary Table 4. Association between PAA as residuals and breast cancer treatment pattern (*N*=1,236).

Treatment	*N*	Year 1		Year 2		Year 5		Year 10	
		Δ PAA	*p*	Δ PAA	*p*	Δ PAA	*p*	Δ PAA	*p*
**Treatment combination[1]**									
Surgery(+radiation)	116	Reference		Reference		Reference		Reference	
Surgery(+radiation)+chemo	171	**1.54**	**0.02**	-0.49	0.329	**-0.98**	**0.023**	0.25	0.617
Surgery(+radiation)+endocrine	326	0.31	0.633	-0.81	0.085	-0.31	0.435	**1.69**	**<0.001**
Surgery(+radiation)+chemo+endocrine	327	0.6	0.336	**-1.29**	**0.006**	**-1.23**	**0.002**	0.7	0.117
Surgery(+radiation)+chemo+endocrine +targeted	106	0.8	0.211	-0.88	0.079	**-1.11**	**0.013**	0.99	0.085
Other	190	0.58	0.358	-0.3	0.529	0.48	0.234	**1.61**	**0.001**
**Medication[2]**	*N* (yes/no)								
Chemotherapy									
Alkylating	634/602	**0.81**	**<0.001**	-0.2	0.192	**-1.02**	**<0.001**	**-0.76**	**<0.001**
Anthracyclines	469/767	**0.67**	**<0.001**	-0.17	0.251	**-1.38**	**<0.001**	**-1.09**	**<0.001**
Antimetabolite	157/1,079	**-2.33**	**<0.001**	0.26	0.096	**1.62**	**<0.001**	**0.8**	**<0.001**
Anti-microtubule/taxane	645/591	-0.05	0.821	**-0.46**	**0.007**	**-0.46**	**0.005**	-0.23	0.25
Endocrine therapy									
SERM	437/799	**-0.35**	**0.049**	**-0.31**	**0.028**	**-0.38**	**0.006**	0.09	0.645
Aromatase inhibitor	634/602	**-0.56**	**0.001**	**-0.63**	**<0.001**	0	0.975	**0.84**	**<0.001**

[1]Difference in PAA estimated using linear mixed models with an interaction between exposures and a natural spline of year since diagnosis, with adjustment for cancer stage, grade, and subtype; [2]Difference in PAA estimated using linear mixed models with an interaction between exposures and a natural spline of year since diagnosis, with adjustment for cancer stage, grade, subtype, other treatments, and other drugs of the treatment type.
